# Worse Survival in Elderly Patients with Extremity Soft-Tissue Sarcoma

**DOI:** 10.1245/s10434-016-5158-7

**Published:** 2016-03-08

**Authors:** Miriam L. Hoven-Gondrie, Esther Bastiaannet, Vincent K.Y. Ho, Barbara L. van Leeuwen, Gerrit-Jan Liefers, Harald J. Hoekstra, Albert J. H. Suurmeijer

**Affiliations:** Department of Surgery, University of Groningen, University Medical Center Groningen, Groningen, The Netherlands; Department of Surgery, University of Leiden, Leiden University Medical Center, Leiden, The Netherlands; Department of Gerontology and Geriatrics, University of Leiden, Leiden University Medical Center, Leiden, The Netherlands; Comprehensive Cancer Center Netherlands, Utrecht, The Netherlands; Department of Pathology, University of Groningen, University Medical Center Groningen, Groningen, The Netherlands

## Abstract

**Background:**

Nearly half of soft-tissue sarcoma (STS) patients are over the age of 65, and the behavior of cancer in these elderly patients is poorly understood. The aim of this study was to assess the impact of age, sarcoma histotype, grade, stage, and treatment modalities on survival of extremity STS (ESTS) patients.

**Methods:**

Patients ≥18 years diagnosed with ESTS between 1989 and 2008 were selected from the Netherlands Cancer Registry. Survival rates and patient and treatment characteristics were analyzed for all patients. Relative survival and relative excess risk of death were estimated for young (<65 years) and older (>65 years) patients.

**Results:**

Overall, 3066 patients were included in this study. Histotype was different between young (<65 years) and elderly (>65 years) patients (*p* < 0.001). Patients over the age of 65 were more often diagnosed with high-stage ESTS and an increasing proportion of high-grade ESTS (*p* < 0.001). The proportion of patients who received no treatment increased with age, and the elderly received fewer combined-modality treatments. Age was significantly associated with relative 5-year survival [72.7 % for younger patients and 43.8 % for the oldest elderly (>85 years)]. In multivariable analysis, age still remained a significant prognostic factor.

**Conclusions:**

Different distribution of sarcoma histotypes, more high-stage and high-grade sarcomas at diagnosis, less aggressive treatment, and worse survival rates emphasize the need for optimizing sarcoma research and care of the elderly.

**Electronic supplementary material:**

The online version of this article (doi:10.1245/s10434-016-5158-7) contains supplementary material, which is available to authorized users.

Soft-tissue sarcomas (STS) are relatively rare tumors and account for 1 % of all cancers in adults.^1^ The overall incidence of STS in the Netherlands is 3.2 per 100,000 (European Standardized Rate 2015).^2^ There has been a slight increase in the incidence of STS in men.^2^ Nearly 50 % of the diagnosed patients are over 65 years of age.^3^ 50–60 % of STS are localized in the extremities. The most common sarcoma histotypes are pleomorphic undifferentiated sarcoma (malignant fibrous histiocytoma), liposarcoma, leiomyosarcoma, and fibrosarcoma.^3^,^4^ Important prognostic factors for STS are primary site, tumor size, histotype, grade, and stage at presentation.^4^ Age at presentation determines survival in several distinct STS histotypes, although the exact cutoff for older age varies in the studies from 45 to 65 years.^5^–^10^

The behavior of cancer in elderly patients is poorly understood.^11^,^12^ There is a widespread misconception that the elderly are poorly tolerant of chemotherapy and radiotherapy.^11^,^12^ In daily clinical practice, elderly patients may be undertreated compared to young or middle-aged STS patients, and this may influence their prognosis.^3^,^13^–^15^ Moreover, elderly patients are generally underrepresented or excluded in cancer trials, which compromises our knowledge of the effectiveness of sarcoma treatment for this age group.^16^ For extremity STS (ESTS), there is a well-defined role for surgery and radiotherapy.^17^ In ESTS removed with narrow margins or microscopically (R1) or macroscopically (R2) positive resection margins, 50–70 Gy of external-beam radiotherapy is indicated.^18^,^19^

 The negative impact of older age on sarcoma care and mortality has been discussed in previous literature. In this study, we focus on ESTS patients. The goal of the study was to assess impact of age on relative survival of adult ESTS patients diagnosed in the Netherlands. Our hypothesis was that older patients were diagnosed at higher stage, underwent less aggressive treatment, and had lower survival rates compared to younger patients.

## Methods

 Patients (age 18 and older) diagnosed with ESTS as first primary malignancy in the period 1989–2008 were selected from the Netherlands Cancer Registry (NCR). Patients with Kaposi sarcoma, dermatofibrosarcoma protuberans, Ewing sarcoma, and a two rare sarcomas (alveolar soft part sarcoma and clear cell sarcoma) were excluded (Supplementary Table S1). There were no other exclusion criteria (population-based dataset). PALGA (Pathologisch-Anatomisch Landelijk Geautomatiseerd Archief), a nationwide network and registry of histopathology and cytopathology diagnosis in the Netherlands, regularly submits reports of all diagnosed malignancies to the cancer registries.^20^ The national hospital discharge data bank, which receives discharge diagnoses of admitted patients from all Dutch hospitals, completed case ascertainment, so that all cancer patients are included. After notification, trained registry personnel collected data on diagnosis, stage, and treatment from the medical records, including pathology and surgery reports, using the registration and coding manual of the NCR for all items. Treatment was coded in sequence of administration and could consist of no treatment, surgery, radiotherapy, or chemotherapy as the only therapy, or a combination (surgery and chemotherapy, surgery and radiotherapy, or surgery and radiotherapy and chemotherapy). Stage was assigned according to the American Joint Committee on Cancer and grade according to the FNCLCC (Fédération Nationale des Centres de Lutte Contre le Cancer).^21^,^22^ Survival status (dead or alive) was established either directly from the patient’s medical record or through linkage of cancer registry data with the municipal population registries, which recorded information on their inhabitants’ survival status (deceased or alive). We used the World Health Organization (WHO) Classification of Soft Tissue Sarcoma for histopathologic classification of sarcoma histotypes.^1^ If necessary, STS were reclassified according to the most recent WHO classification (2013) (Supplementary Table S1).

Differences in stage at diagnosis, sarcoma histotype, tumor grade, and treatment regimens were compared between young and elderly sarcoma patients. Age was categorized in the following groups: patients <65 and elderly patients >65 years, further subdivided into 5-year cohorts: 65–69, 70–74, 75–79, 80–84, and 85+ years. Stage at diagnosis was compared according to age categories. Treatment was categorized into no treatment, monotherapy (surgery, radiotherapy, chemotherapy), and combinations thereof (surgery and chemotherapy, surgery and radiotherapy or surgery, radiotherapy and chemotherapy). Relative survival was calculated as cause of death was not known for these patients. Relative survival was calculated as the ratio of the survival observed among the ESTS patients and the survival that would have been expected based on the corresponding (age, sex, and year) general population. Relative excess risk (RER) of death was estimated using a multivariable generalized linear model with a Poisson error structure, based on collapsed relative survival data, using exact survival times. Survival time was calculated from the date of diagnosis and ended at the date of death, date of last contact, or date of most recent linkage with the municipal population registries, whichever came first. Relative mortality (as a result of ESTS) was calculated by the following equation: [(observed deaths − expected deaths)/observed deaths]. National life tables were used to estimate expected survival. Stata/SE 12.0 software (StataCorp, College Station, TX) was used to perform statistical analyses.

## Results

As shown in Table 1, 3066 patients were included in this study. At first diagnosis, nearly 40 % of patients were aged 65 years or older, two-thirds (69 %) had high-grade sarcomas, and 71 % were diagnosed with stage I or II disease. As shown in Table 1, there were significant differences in distribution of sarcoma histotypes in young (<65 years) versus elderly (>65 years) patients (*p* < 0.001). Using 65 years of age as the cutoff value, myxoid and round cell liposarcoma were diagnosed more often in younger patients (*p* < 0.0001), whereas pleomorphic undifferentiated sarcoma was recorded more often in the elderly (*p* < 0.0001). Leiomyosarcoma was predominantly diagnosed in the 70–74 age group (*p* < 0.001). Elderly patients presented with high-grade disease more often, ranging from 66 % high-grade disease in patients younger than 65–81 % in the 85+ age group (*p* < 0.001).

**Table 1:** Characteristics of population by age category

Characteristic	Variable	Age						p*
		<65	65–69	70–74	75–79	80–84	85+	
Sex	Male	1009 (54.0)	145 (50.5)	142 (52.2)	148 (55.6)	109 (53.4)	92 (55.1)	0.8
	Female	861 (46.0)	142 (49.5)	130 (47.8)	118 (44.4)	95 (46.6)	75 (44.9)	
Year	1989–1993	448 (24.0)	47 (16.4)	69 (25.4)	55 (20.7)	49 (24.0)	34 (20.4)	0.2
	1994–1998	416 (22.3)	77 (26.8)	71 (26.1)	58 (21.8)	54 (26.5)	36 (21.5)	
	1999–2003	474 (25.3)	72 (25.1)	68 (25.0)	68 (25.6)	51 (25.0)	45 (27.0)	
	2004–2008	532 (28.4)	91 (31.7)	64 (23.5)	85 (31.9)	50 (24.5)	52 (31.1)	
Grade	Low grade	636 (34.0)	77 (26.8)	70 (25.7)	61 (22.9)	46 (22.6)	32 (19.2)	<0.001
	High grade	1234 (66.0)	210 (73.2)	202 (74.3)	205 (77.1)	158 (77.4)	135 (80.8)	
Sarcoma	Dedifferentiated liposarcoma	8 (0.4)	3 (1.1)	1 (0.4)	4 (1.5)	3 (1.5)	3 (1.8)	0.08
	Myxoid and round cell liposarcoma	335 (17.9)	17 (5.9)	17 (6.3)	9 (3.4)	4 (2.0)	7 (4.2)	<0.001
	Pleomorphic liposarcoma	53 (2.8)	7 (2.4)	9 (3.3)	5 (1.9)	8 (3.9)	5 (3.0)	0.8
	Mixed-type liposarcoma	22 (1.2)	2 (0.7)	1 (0.4)	3 (1.1)	1 (0.5)	1 (0.6)	0.7
	Fibrosarcoma	194 (10.4)	37 (12.9)	24 (8.8)	32 (12.0)	24 (11.8)	19 (11.4)	0.6
	Pleomorphic undifferentiated sarcoma	521 (27.9)	113 (39.4)	114 (41.9)	121 (45.5)	101 (49.5)	85 (50.9)	<0.001
	Leiomyosarcoma	339 (18.1)	78 (27.2)	80 (29.4)	74 (27.8)	46 (22.6)	27 (16.2)	<0.001
	Rhabdomyosarcoma	55 (2.9)	8 (2.8)	4 (1.5)	3 (1.1)	8 (3.9)	6 (3.6)	0.3
	Angiosarcoma	29 (1.6)	6 (2.1)	4 (1.5)	4 (1.5)	5 (2.4)	9 (5.4)	0.002
	Synovial sarcoma	228 (12.2)	10 (3.5)	14 (5.1)	7 (2.6)	2 (1.0)	1 (0.6)	<0.001
	Malignant peripheral nerve sheath tumor	86 (4.6)	6 (2.1)	4 (1.5)	4 (1.5)	2 (1.0)	4 (2.4)	0.002
	Overall							<0.001**

* *p* value (*χ* ^2^) for differences in distribution across age categories

** Overall difference in sarcoma distribution across age categories

**Fig. 1:**
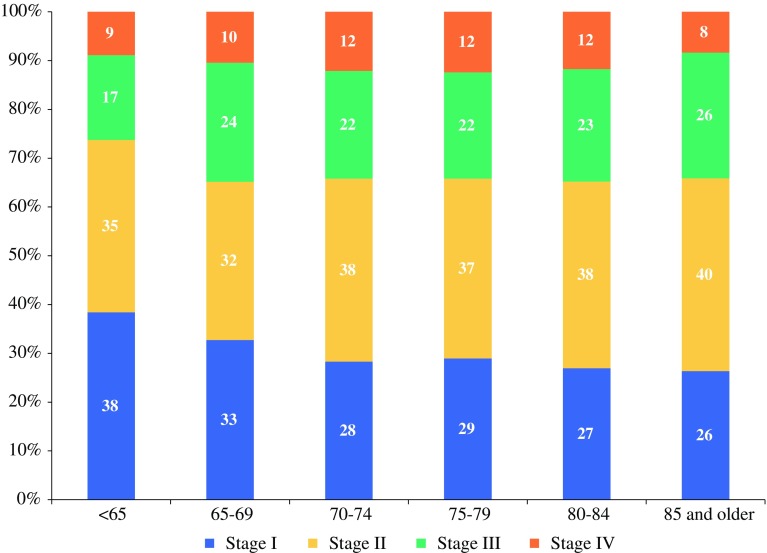
Stage at diagnosis according to age group

Stage at diagnosis was different between age categories (*p* < 0.001, Fig. 1). The relative proportion of stage I disease diminished from 38 % in patients <65 years to 27 % for the 85+ patients. High-stage disease (stages III and IV) was more prominent among patients >65 years: 34 % compared to 26 % in patients younger than 65.

As shown in Table 2, the proportion of patients who received no treatment was higher among patients >65 years and particularly patients older than 80. Overall, for patients over the age of 80 years (*n* = 371), 14.8 % (*n* = 55) received no treatment compared to 2.7 % of patients <65 (*p* < 0.001). Surgery as sole treatment was administered more often to the elderly (ranging from 42 % in patients <65 years to 53 % in patients 85+ years; *p* = 0.002). A combination of surgery and radiotherapy was provided less often to the elderly, particularly patients 85+ years (40 % of patients younger than 65 compared to 27 % of the 85+ patients; *p* = 0.004).

**Table 2:** Treatment for extremity soft tissue sarcoma patients

Treatment	Age	*n* (%)	OR (95 % CI)	*p*	Adjusted OR (95 % CI)	p*
No treatment				<0.001		<0.001
	<65	50 (2.7)	1.0 (ref)		1.0 (ref)	
	65–69	13 (4.5)	1.7 (0.9–3.2)		1.6 (0.8–3.1)	
	70–74	24 (8.8)	3.5 (2.1–5.8)		3.2 (1.8–5.6)	
	75–79	15 (5.6)	2.2 (1.2–3.9)		1.7 (0.9–3.3)	
	80–84	28 (13.7)	5.8 (3.6–9.4)		5.6 (3.2–9.6)	
	85+	27 (16.2)	7.0 (4.3–11.6)		8.3 (4.7–14.6)	
Surgery (monotherapy)				0.002		0.0003
	<65	787 (42.1)	1.0 (ref)		1.0 (ref)	
	65–69	105 (36.6)	0.8 (0.6–1.0)		0.8 (0.6–1.0)	
	70–74	93 (34.2)	0.7 (0.5–0.9)		0.7 (0.5–0.9)	
	75–79	121 (45.5)	1.1 (0.9–1.5)		1.2 (0.9–1.6)	
	80–84	82 (40.2)	0.9 (0.7–1.2)		0.9 (0.7–1.3)	
	85+	88 (52.7)	1.5 (1.1–2.1)		1.7 (1.2–2.3)	
Radiotherapy (monotherapy)				<0.001		0.0003
	<65	13 (0.7)	1.0 (ref)		1.0 (ref)	
	65–69	4 (1.4)	2.0 (0.7–6.2)		2.0 (0.6–6.2)	
	70–74	5 (1.8)	2.7 (0.9–7.6)		2.4 (0.8–7.0)	
	75–79	12 (4.5)	6.7 (3.0–15.0)		6.3 (2.7–14.8)	
	80–84	9 (4.4)	6.6 (2.8–15.6)		6.1 (2.4–15.1)	
	85+	3 (1.8)	2.6 (0.7–9.3)		2.4 (0.7–9.0)	
Chemotherapy (monotherapy)				0.4		0.2
	<65	74 (4.0)	1.0 (ref)		1.0 (ref)	
	65–69	13 (4.5)	1.2 (0.6–2.1)		1.1 (0.6–2.2)	
	70–74	14 (5.2)	1.3 (0.7–2.4)		1.2 (0.7–2.4)	
	75–79	7 (2.6)	0.7 (0.3–1.4)		0.5 (0.2–1.2)	
	80–84	4 (2.0)	0.5 (0.2–1.3)		0.4 (0.1–1.1)	
	85+	4 (2.4)	0.6 (0.2–1.6)		0.7 (0.2–2.0)	
Surgery and chemotherapy				0.06		0.08
	<65	96 (5.1)	1.0 (ref)		1.0 (ref)	
	65–69	16 (5.6)	1.1 (0.6–1.9)		1.1 (0.6–2.0)	
	70–74	8 (2.9)	0.6 (0.3–1.2)		0.6 (0.3–1.2)	
	75–79	5 (1.9)	0.4 (0.1–0.9)		0.4 (0.1–0.9)	
	80–84	6 (2.9)	0.6 (0.2–1.3)		0.5 (0.2–1.3)	
	85+	0 (0.0)	–		–	
Surgery and radiotherapy				0.004		0.0005
	<65	739 (39.5)	1.0 (ref)		1.0 (ref)	
	65–69	124 (43.2)	1.2 (0.9–1.5)		1.2 (1.0–1.6)	
	70–74	124 (45.6)	1.3 (1.0–1.6)		1.4 (1.1–1.9)	
	75–79	102 (38.4)	1.0 (0.7–1.2)		1.0 (0.7–1.3)	
	80–84	74 (36.3)	0.9 (0.6–1.2)		0.9 (0.7–1.3)	
	85+	45 (27.0)	0.6 (0.4–0.8)		0.5 (0.4–0.8)	
Surgery and radiotherapy and chemotherapy				0.001		0.003
	<65	96 (5.1)	1.0 (ref)		1.0 (ref)	
	65–69	10 (3.5)	0.7 (0.4–1.3)		0.7 (0.3–1.3)	
	70–74	4 (1.5)	0.3 (0.1–0.8)		0.3 (0.1–0.8)	
	75–79	4 (1.5)	0.3 (0.1–0.8)		0.3 (0.1–0.8)	
	80–84	1 (0.5)	0.1 (0.01–0.7)		0.1 (0.01–0.7)	
	85+	0 (0.0)	–		–	

Chemotherapy and radiotherapy: 15 patients <65 years and two patients aged 65–69 years

*Ref* reference category

* *p* value adjusted for sex, year, grade, stage, and type of sarcoma

**Fig. 2:**
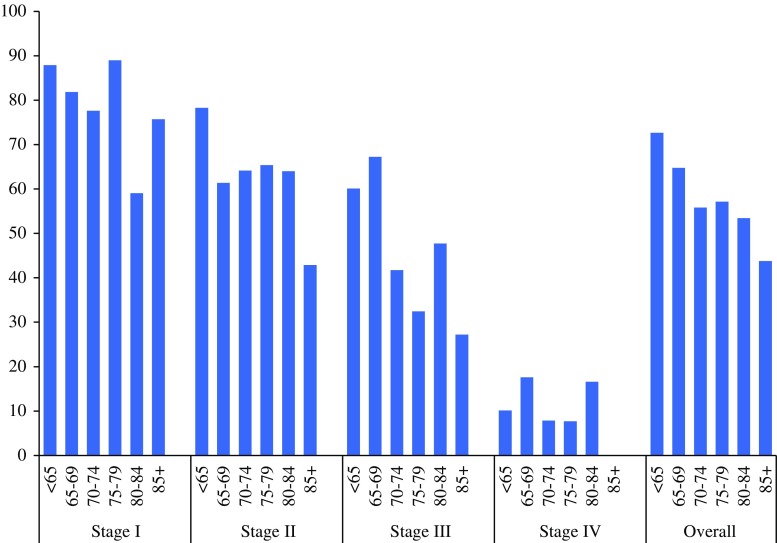
Relative survival for sarcoma patients according to patient age and disease stage

**Fig. 3:**
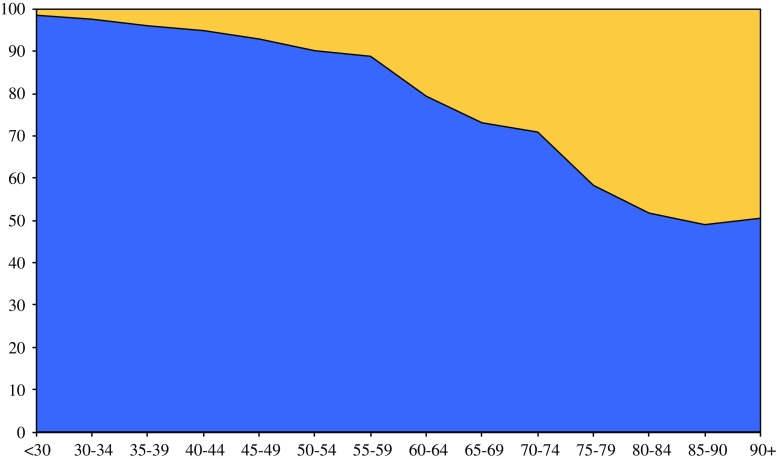
Observed minus expected deaths (10-year follow-up) as proportion of observed deaths in the population of sarcoma patients according to age. *blue* estimated death due to sarcoma; *yellow* estimated death due to other causes

Mean follow-up time was 6.2 years with a median of 4.2 (range 0–21) years. Patients younger than 65 had a 5-year relative survival (all stages) of 72.7 % (95 % confidence interval 70.4–74.8). For patients >65 years the 5-year relative survival was significantly worse, decreasing with age to 43.8 % (95 % confidence interval 28.3–62.3) for the 85+ patients. Stratified for stage and age, the *p* value for trend (age) was *p* = 0.01 for stage I, *p* < 0.001 for stage II, *p* = 0.0001 for stage III, and *p* = 0.02 for stage IV, respectively (Fig. 2). When adjusted for sex, year, sarcoma histotype, grade, and treatment in multivariable analysis, significant differences between the age groups remained (stage I: *p* = 0.02, stage II: *p* = 0.0002, stage III: *p* = 0.0001, stage IV: *p* = 0.028). The results were confirmed after analyzing age as a continuous variable in the multivariable models: in stage I the RER was 1.02 (1.01–1.04) with a *p* value of 0.002, for stage II RER 1.02 (1.01–1.03), *p* < 0.001, for stage III RER 1.02 (1.01–1.02), *p* = 0.001, and for stage IV RER 1.01 (1.00–1.02), *p* = 0.01. Figure 3 shows that the estimated deaths (equation [(observed deaths − expected deaths)/observed deaths]) due to sarcoma after 10 years of follow-up rapidly decreases among the elderly, although the proportion does not decrease below 50 % of all deaths.

## Discussion

Because the elderly population is growing fast and the incidence of STS increases with age, elderly cancer care has become a growing challenge to the world health care systems.^23^ In keeping with most other series, we found worse survival outcomes among elderly ESTS patients.^24^–^26^ Differences in presentation and treatment have often been mentioned as possible explanations for this inferior prognosis.^10^,^25^–^28^

Part of the reason older patients do worse is that they often present with larger and higher-grade tumors.^23^,^26^,^27^ This increased rate of high-grade tumors may reflect a different tumor biology and more genetic alterations and molecular aberrations in the older population.^29^ Age can also be associated with a decline in anti-tumor-cell-mediated immunity.^30^

Concerning sarcoma histotype, more pleomorphic undifferentiated sarcomas are diagnosed in the elderly, which are known to have a relatively adverse prognosis.^25^ Histopathologic classification of soft tissue sarcoma has become more accurate over the past 10 years due to advances in immunohistochemistry (newly developed antibodies) and molecular biology (FISH and PCR detecting newly discovered gene translocations which are more or less histotype specific). Because our study included cases studied between 1989 and 2008, this may have led to an overrepresentation of the percentage of pleomorphic undifferentiated sarcomas, for instance. For prognosis, treatment, or response to treatment, molecular characterization already has clinical implications for some subtypes of sarcoma, and new targets for therapy are increasingly being revealed.^31^,^32^

In this series, elderly patients had less stage I disease at presentation. This may be a function of late presentation, often seen in elderly patients with malignancy.^27^ However, after stratifying for stages, there are still significant differences in relative survival between young and elderly ESTS patients. This survival difference was found mainly in stage II and III ESTS patients. A possible explanation might be that less extensive treatment regimens often suffice in patients with stage I disease, so age will not be the reason to withhold any patient from optimal treatment. For stage IV disease, we know that overall prognosis is poor for all patients, regardless of the extent of treatment. This leads to the speculation that suboptimal treatment in the elderly might contribute to worse survival rates in stage II and III ESTS patients. A former study from the cancer registry in the northern part of the Netherlands showed that referral to a specialized center declined in a linear fashion with increased age, which might explain this possible suboptimal treatment of elderly patients.^13^ Postgraduate medical training of general practitioners, nursing home physicians, and geriatricians in the differential diagnosis of soft-tissue masses might contribute to early and adequate referral.

The principles of treatment for elderly patients are similar to those for younger patients and those described in our national guidelines.^33^ For most ESTS, a combined treatment modality is assumed to be the optimal therapy and will be offered to every patient fit enough to undergo this treatment.^15^

The mainstay of ESTS treatment is surgery.^34^ In a proportion of elderly patients, single treatment (e.g. radical surgery) is the best option, even if this treatment is suboptimal with respect to local or systemic control of sarcoma.^34^ Surgeons might be reluctant to perform, or patients to accept, morbid surgeries with increasing patient age, leading to inferior surgical treatment.^26^,^35^ In our series, almost 90 % of patients underwent any form of surgery. In the cohort of patients >75 years, this proportion was still 83 %. Lahat and colleagues have already advocated radical surgery in the elderly, as properly selected patients can safely undergo extensive STS resections. Their study showed that even in the group of patients aged ≥75, more than half survived 5 years or more when treated with aggressive surgery.^23^ However, a large but highly select group of patients was investigated in this series. Patients with incomplete resection, insufficient follow-up, and nonspecific pathologic diagnoses were excluded; this might be a patient group with unfavorable prognostic characteristics but one that could actually benefit from better staging, referral, or customized treatment. Buchner et al. also justify extensive surgery in bone and soft tissue sarcoma patients aged >70. They state, however, that general condition and comorbidity should be given due consideration.^36^ Fitness for general anesthesia should be the determinant for suitability for surgery in elderly patients rather than chronological age.^37^

Patients with gross disease will benefit from preoperative radiotherapy, although this is accompanied with increased treatment-related morbidity such as wound-healing disturbances and those with less than adequate margins after surgery from adjuvant radiotherapy.^34^,^38^ In particular, surgery combined with radiotherapy was less often administered in the 85+ group compared to the other age groups. Physicians can be cautious in offering (neo-)adjuvant radiotherapy to elderly patients, especially if there is an expected benefit in the longer term while the overall life expectancy might be short. Furthermore, logistical problems and patient preferences are often the reason that elderly patients refuse long courses of external radiation.^27^ Horton et al. showed that patients aged ≥70 with high-grade ESTS were less likely to receive radiotherapy, and furthermore that not receiving radiotherapy was associated with mortality and disease-specific death.^39^ In the population-based study of Al-Refaie et al., sarcoma-directed surgery and administration of adjuvant radiotherapy after limb-sparing surgery for T2 or high-grade tumors were also decreased for ESTS patients aged ≥85.^28^

The role of chemotherapy in the treatment of ESTS remains controversial. Chemotherapy turns out to be ineffective in the majority of the ESTS, and toxicity may be significant.^34^,^40^,^41^ As expected, administration of chemotherapy with or without surgery and/or radiotherapy declines with age in our series. There is, however, a widespread misconception that the elderly are always poorly tolerant of chemotherapy.^11^,^42^ Most adult cancer patients can tolerate it with limited impact on independence, comorbidity, and quality-of-life levels, although half of the population experienced severe adverse effects.^42^

The proportion of patients who received no treatment at all increased significantly with age. Former studies from our own center showed that the decision to refrain from cancer treatment was mostly disease related and was less often based on poor general health status.^15^

Strikingly, in contrast to most other types of cancer, and despite continuous efforts to improve sarcoma treatment, more individual customized therapy, relatively new treatment modalities (like hyperthermic isolated limb perfusion for locally advanced STS of the extremity), and better compliance with guidelines, other series show that survival rates have not improved over time for all age groups.^43^ Further research is thus extremely important to discover the gaps in sarcoma treatment and find areas with room for improvement.

The results of this study are based on data from a cancer registry, which was used in order to gain insight into the disease and discover differences in treatment and survival for different age groups. Because there are no data on patient frailty in the cancer registry, we cannot comment on the just or unjust allocation of treatment. Accurate reports in the medical records on diagnosis, staging, treatment, and follow-up details are essential for reliable registration and analysis. Because the national cancer registry does not register for comorbidity, type of surgery and/or chemotherapy, quality of surgery, or hospital-level factors, a more detailed analysis was not possible.

Elderly patients are also generally underrepresented in cancer trials, which compromises the generalizability of the effectiveness of sarcoma treatments to this age group, and future efforts should identify methods to improve low accrual rates of the elderly in clinical cancer trials and should explore other research designs to study the elderly.^16^,^44^

## Conclusions

Studies on STS care in the elderly are extremely important to gain better insight into treatment and survival differences; the current norm of individual, customized sarcoma treatment also applies to elderly ESTS patients. Different distribution of sarcomas, more high-grade and high-stage sarcomas, less aggressive treatment, and lower survival of the elderly emphasize the need for optimizing sarcoma care that may improve survival rates of elderly sarcoma patients.

## Electronic supplementary material

Below is the link to the electronic supplementary material.

Supplementary material 1 (DOCX 14 kb)
