# Supplementary material for: Worse Survival in Elderly Patients with Extremity Soft-Tissue Sarcoma
Source: Ann Surg Oncol. 2016 Mar 8;23:2577–85. doi: 10.1245/s10434-016-5158-7 (PMC4927613; doi:10.1245/s10434-016-5158-7)
Supplement: Supplementary file 1 — Supplementary material 1 (DOCX 14 kb) [file 10434_2016_5158_MOESM1_ESM.docx]

Attachment 1: Morphology codes included in the selection of patients with soft-tissue sarcoma

| **Group** | **Morphology (WHO 2002)** | **Number** |
| --- | --- | --- |
| Dedifferentiated liposarcoma | 8858 | 22 |
| Myxoid & round cell liposarcoma | 8852 & 8853 | 389 |
| Pleomorphic liposarcoma | 8854 | 87 |
| Mixed-type liposarcoma | 8855 | 30 |
| Fibrosarcoma | 8825, 8811, 8814, 8810, 8812 & 8813 | 330 |
| Pleomorphic undifferentiated sarcoma | 8830 & 8800-8809 | 1055 |
| Leiomyosarcoma | 8890, 8891 & 8896 | 644 |
| Rhabdomyosarcoma | 8900-8902, 8910, 8912, 8920 & 8921 | 84 |
| Angiosarcoma | 9120 | 57 |
| Synovial sarcoma | 9040-9043 | 262 |
| MPNST | 9540 | 106 |
| Overall | All | 3066 |
